# Evaluation of Latent Membrane Protein 1 as a novel vaccine adjuvant

**DOI:** 10.1186/1742-4690-9-S2-P11

**Published:** 2012-09-13

**Authors:** JM Termini, S Gupta, GW Stone

**Affiliations:** 1 University of Miami, Miami, FL, USA

## Background

The EBV protein Latent Membrane Protein-1 (LMP1) is known to constitutively activate B cells. The LMP1 signaling pathway mimics that of CD40, a molecule involved in dendritic cell activation and maturation. Therefore we decided to evaluate the use of LMP1 as a vaccine adjuvant for both dendritic cell therapeutic vaccines and DNA-based vaccines for HIV.

## Methods

To determine activity, LMP1 was analyzed using a luciferase report assay for NF-kB and IFN-β. To establish if LMP1 could activate human monocyte-derived dendritic cells (DC), LMP1 transfected DC were analyzed for activation/maturation markers and cytokines. DC migration was determined using a transwell-migration assay. LMP1 was also evaluated in a DNA vaccination/flu challenge mouse model. To determine the benefits of incorporating LMP1 into a DC therapeutic vaccine, LMP1 was tested in a tumor DC therapy mouse model.

## Results

LMP1 activated high levels of NF-kB and IFN-β when evaluated using a luciferase reported assay. On primary DC, LMP1 induced DC activation, maturation, and proinflammatory cytokines. LMP1 induced 2-fold higher migration rates compared to the mature-DC control. As a DNA vaccine for flu, the addition of LMP1 provided superior TNF-α and IFN-γ responses. LMP1 vaccinated animals cleared virus more quickly and in the high-dose lethal flu challenge, LMP1 afforded more protection. Finally, LMP1 enhanced a DC therapeutic vaccine in a tumor model. Tumor progression was slowed compared to antigen-loaded DC alone and positive control mimic-matured DC.

## Conclusion

These data suggest that LMP1 is an effective vaccine adjuvant. LMP1 can enhance the activation, maturation, and functional activity of DC. LMP1 can inducing a strong CD8+ T cell response in several mouse models, most notably the flu viral challenge model. LMP1 increased antigen-specific CD8+ T cells, improved survival to lethal flu high-dose challenge, and slowed tumor progression. These results suggest that LMP1 is a promising adjuvant for prophylactic vaccines for HIV.

